# Supplementary material for: In silico studies provide new structural insights into trans-dimerization of β1 and β2 subunits of the Na+, K+-ATPase
Source: PLoS One. 2025 Apr 29;20(4):e0321064. doi: 10.1371/journal.pone.0321064 (PMC12040271; doi:10.1371/journal.pone.0321064)
Supplement: S1 Table — (DOCX) [file pone.0321064.s001.docx]

**Table S1.** Interactions in the interface calculated in the different conformations of ATP1B1 and ATP1B2

| PDB Pisa structure | **Percentage of Interface residues** | | | | | | |
| --- | --- | --- | --- | --- | --- | --- | --- |
| **Protein** | **Conformation Obtained from MD simulation** | | | | | | |
| **ATP1B1** | 0ns | 20ns | 60ns | 100 ns | 120ns | 160 ns | 170 ns |
| Chain A | 13.3% | 5.4 % | 8.8% | 5.8% | 7.9% | 7.9% | 7.5% |
| Chain B | 12.1% | 5.8% | 8.3% | 5.8% | 6.7% | 5.4% | 5.4% |
| **ATP1B2** | 0ns | 20ns | 60ns | 100 ns | 120ns | 160 ns | 170 ns |
| Chain A | 10.6% | 5.5 % | 6.9% | 11.5% | 8.8% | 9.2% | 7.4% |
| Chain B | 10.4% | 6.5% | 6.9% | 9.7% | 9.2% | 10.1% | 6.0% |
