# Supplementary material for: In silico studies provide new structural insights into trans-dimerization of β1 and β2 subunits of the Na+, K+-ATPase
Source: PLoS One. 2025 Apr 29;20(4):e0321064. doi: 10.1371/journal.pone.0321064 (PMC12040271; doi:10.1371/journal.pone.0321064)
Supplement: S2 Table — The interaction is expressed as pseudo energy, whose ranges have been standardized using known sets of protein-protein complexes. (DOCX) [file pone.0321064.s002.docx]

**Table S2.** Interactions* in the interface calculated in the different conformations of

β_1_ β_1_ and β_2_ -β_2_

| PPCHECK | Energy of the Interactions in the interface | | | | | | |
| --- | --- | --- | --- | --- | --- | --- | --- |
| **β_1_-β_1_** | **0ns** | **20ns** | **60ns** | **100 ns** | **120ns** | **160ns** | **170 ns** |
| Hydrogen bonds (kJ/mol) | -9.81 | -7.77 | -10.04 | -19.22 | -17.60 | -13.59 | -16.86 |
| Electrostatic energy (kJ/mol) | -12.49 | -14.31 | – 31.94 | -36.42 | - 19.32 | -11.88 | -12.37 |
| Van der Waals (kJ/mol) | -87.19 | -28.09 | -63.6 | -60.33 | -70.07 | -55.09 | -94.19 |
| Total stabilizing energy (kJ/mol) | -109.5 | -50.18 | -105.57 | -115. 96 | -106.98 | -80.56 | -123,42 |
| Normalized energy per residue (kJ/mol) | 1.08 | -0.78 | -1.43 | -2.03 | -1.67 | -1.37 | -2,52 |
| **β_2_-β_2_** | **0ns** | **20ns** | **60ns** | **100 ns** | **120ns** | **160ns** | **170 ns** |
| Hydrogen bonds (kJ/mol) | -10.91 | -14.02 | -9.7 | -11.95 | 0.0 | -11.70 | 0.00 |
| Electrostatic energy (kJ/mol) | 66.98 | 12.36 | - 62 | 3.08 | -43.81 | -34.87 | -4.09 |
| Van der Waals (kJ/mol) | -25.22 | -29.34 | -21.85 | -33.18 | -52.79 | -92.87 | -7.89 |
| Total stabilizing energy (kJ/mol) | 30.84 | -31 | -64.84 | -42.05 | -96.60 | -139.43 | -11.98 |
| Normalized energy per residue (kJ/mol) | 0.33 | -0.72 | -1.10 | -0.55 | -1.40 | -1.81 | -0.21 |

*The interaction is expressed as pseudo energy, whose ranges have been standardized using known sets of protein-protein complexes.
