# Supplementary figures and images for: In silico studies provide new structural insights into trans-dimerization of β1 and β2 subunits of the Na+, K+-ATPase
Source: PLoS One. 2025 Apr 29;20(4):e0321064. doi: 10.1371/journal.pone.0321064 (PMC12040271; doi:10.1371/journal.pone.0321064)

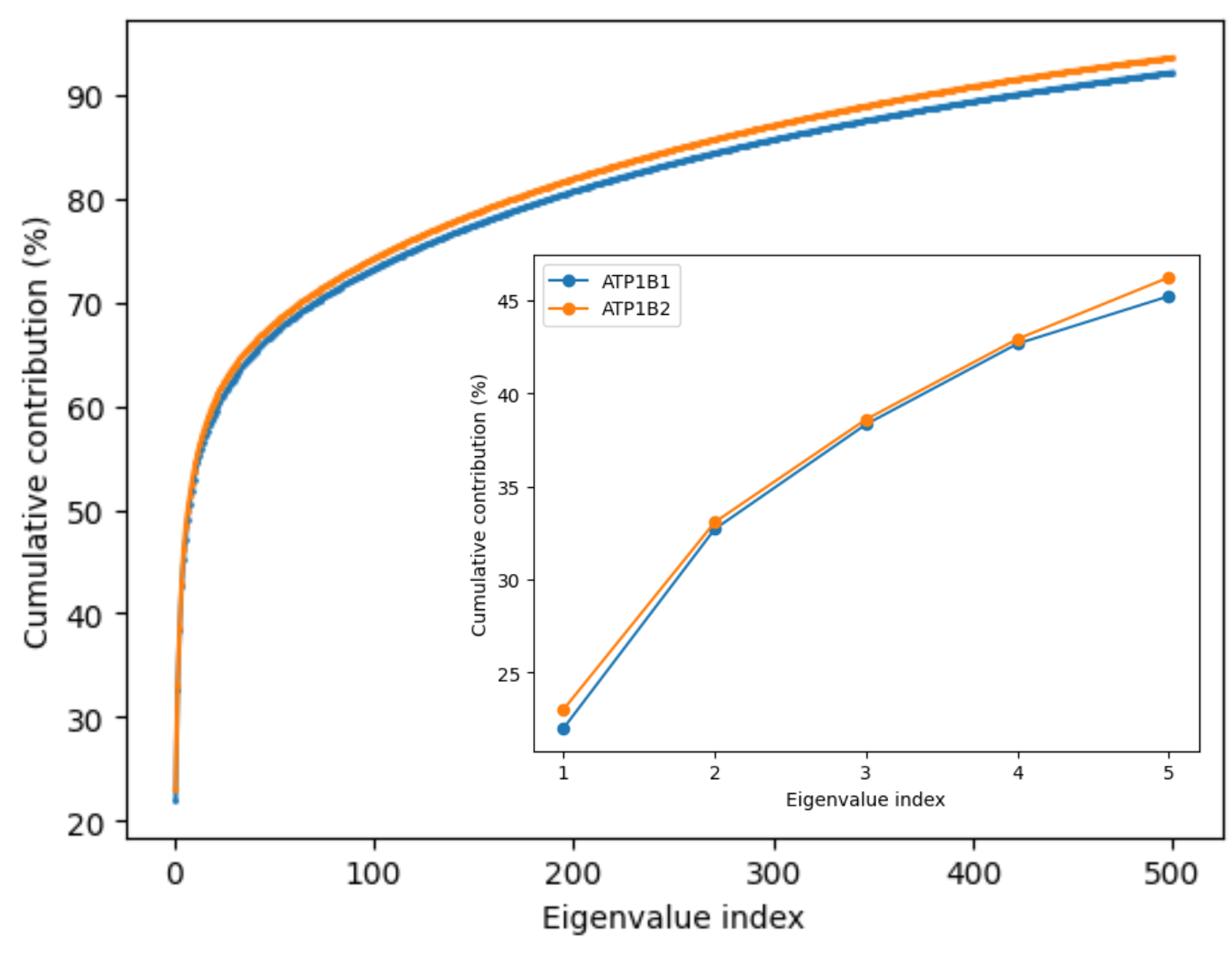

Supplement: S1 Fig — The first principal components for both dimers explain under 40% of the energy observed in the simulation. Reaching 80% requires over 100 principal components, which suggests the simulation time might have to be extended to allow fewer motions to dominate the dynamics. The projections of the trajectories onto PC1 and PC2 are very different, which was expected as the sequences show only partial similarity. Regarding the cluster analysis, some clusters of similar size to the main cluster are observed, which correlates with the poor dominance shown by the main principal components and hints at the possibility of a main energetic basin still waiting to be populated. The motions associated with the two main principal components for ATP1B1 show symmetric, rotatory behaviors that are expected in a stable dimer. In contrast, for ATP1B2, PC1 shows an asymmetric, longitudinal motion that seems to drive the monomers away from each other while PC2 seems to involve mostly inconsequential motions in the most mobile loops. This behavior can be related to Table I, where ATP1B2 shows unfavorable interactions for several of the conformations considered. (TIF) [file pone.0321064.s003.tif]

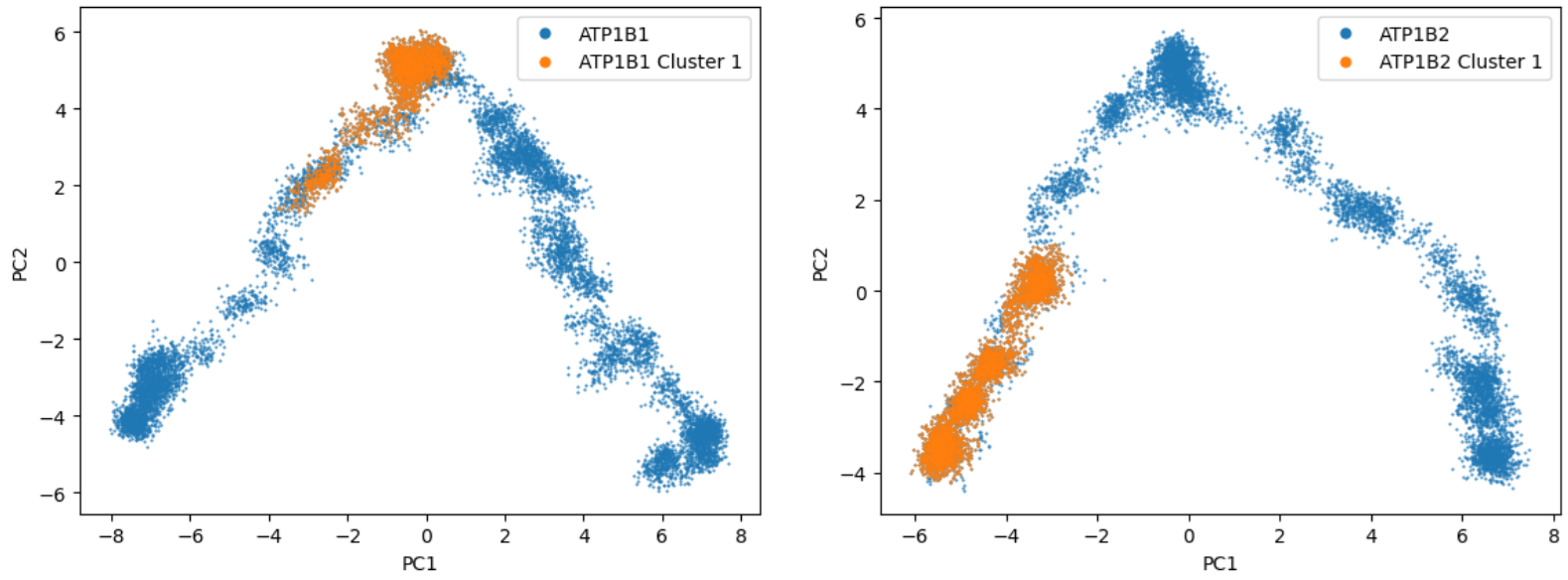

Supplement: S2 Fig — For ATP1B1 we have the main cluster around low values of PC1, whereas the main cluster for ATP1B2 is in a region with large values for both PC1 and PC2, and it also spans a larger region. These observations support the conclusion that ATP1B1 shows a stable interface and the lack thereof for ATP1B2. (TIF) [file pone.0321064.s004.tif]

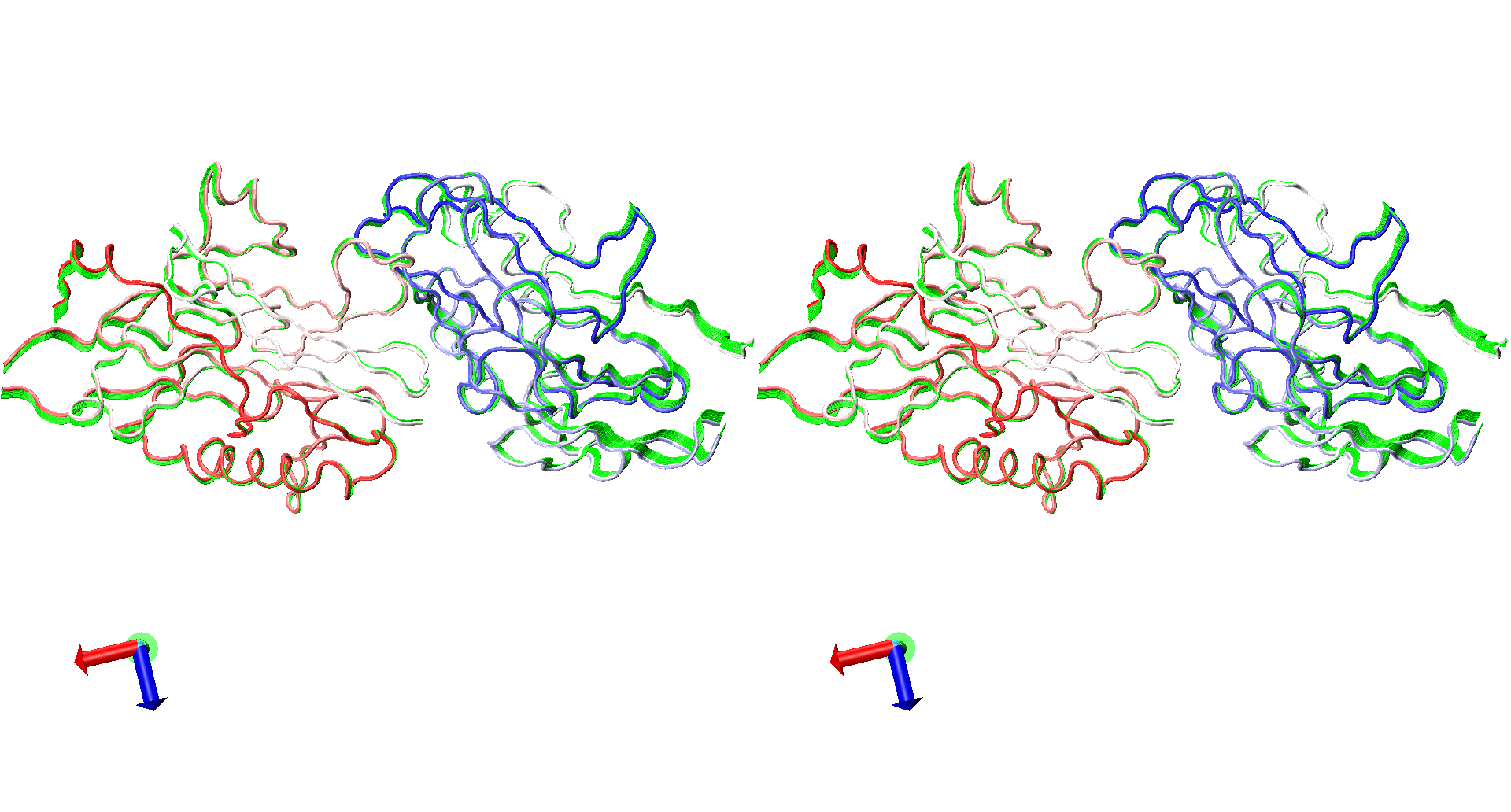

Supplement: S3 Fig — Chain A goes from red in the N-terminus to white in the C-terminus while chain B goes from white in the N-terminus to blue in the C-terminus. Green tubes show the motion associated with the principal components. The motion for PC1 is concerted, symmetric and rotatory, suggesting that a stable interface is reached in the simulation for this dimer. (TIF) [file pone.0321064.s005.tiff]

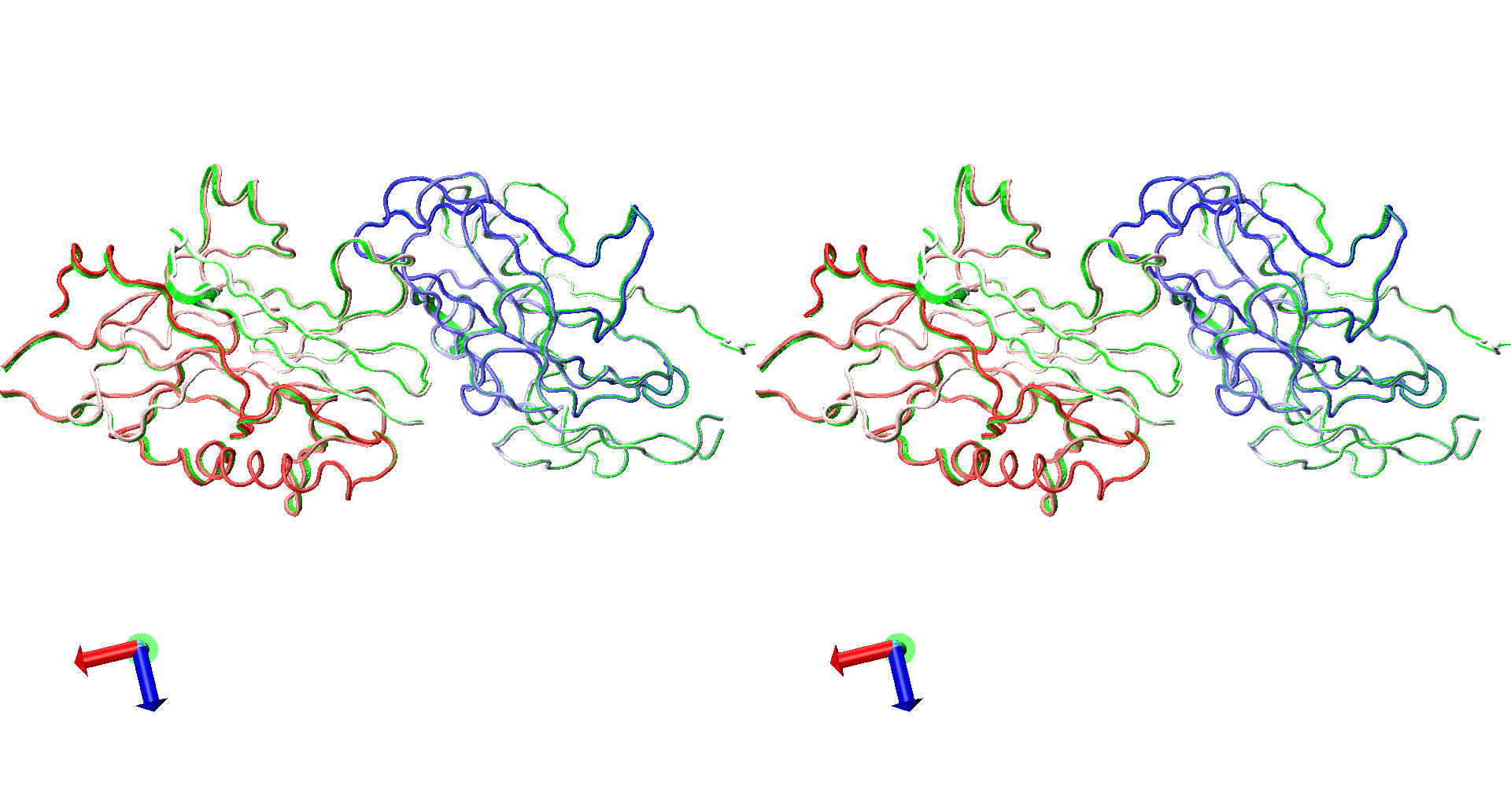

Supplement: S4 Fig — Chain A goes from red in the N-terminus to white in the C-terminus while chain B goes from white in the N-terminus to blue in the C-terminus. Green tubes show the motion associated with the principal components. The motion for PC2 shows less amplitude than the motion for PC1, but is also concerted, symmetric and rotatory, suggesting that a stable interface is reached in the simulation for this dimer. (TIF) [file pone.0321064.s006.tiff]

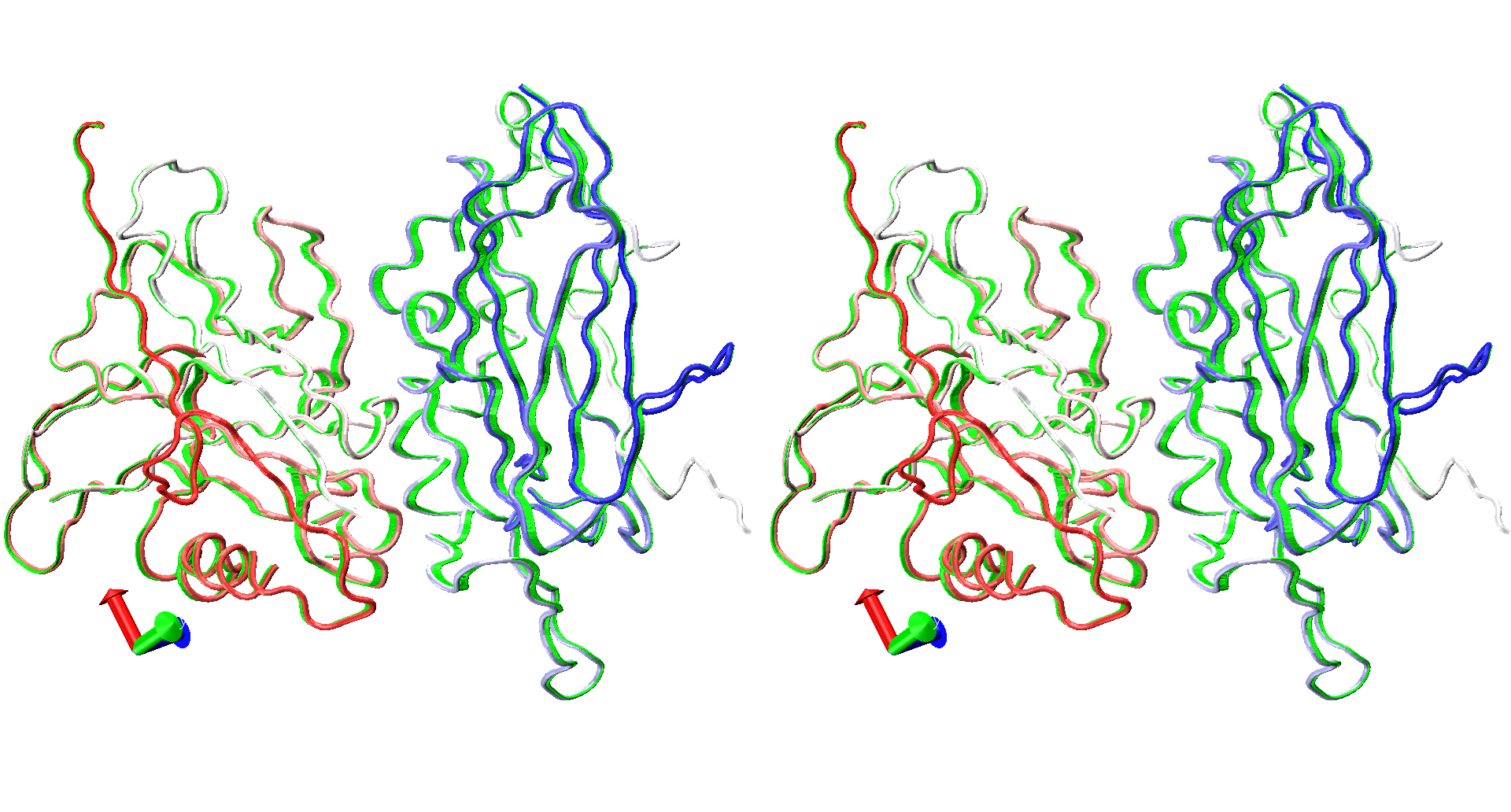

Supplement: S5 Fig — Chain A goes from red in the N-terminus to white in the C-terminus while chain B goes from white in the N-terminus to blue in the C-terminus. Green tubes show the motion associated with the principal components. The motion for PC1 is longitudinal instead of rotatory and shows a tendency to increase the distance between the monomers, which suggests that a stable dimer is not reached in the simulation. (TIF) [file pone.0321064.s007.tiff]

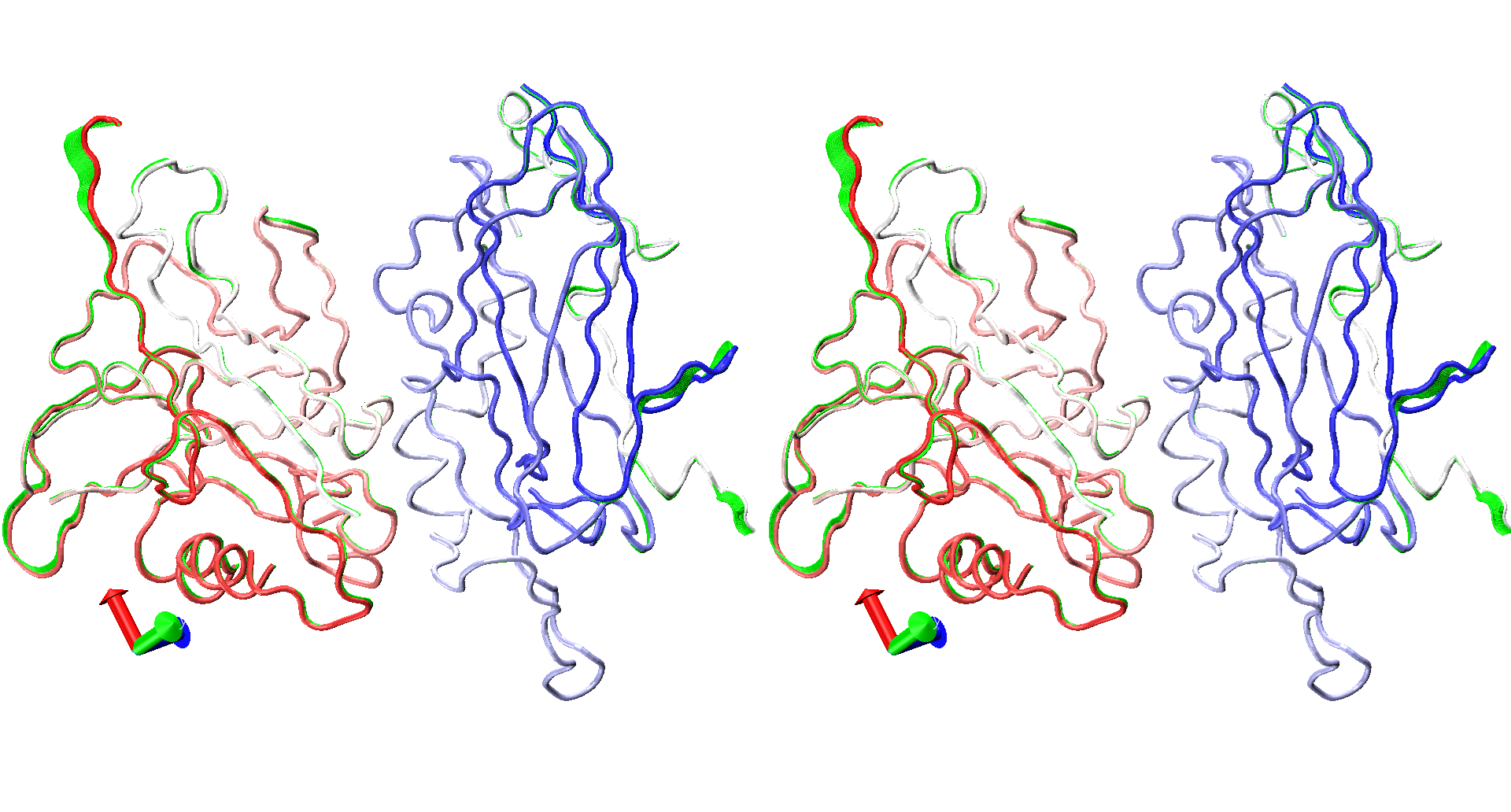

Supplement: S6 Fig — Chain A goes from red in the N-terminus to white in the C-terminus while chain B goes from white in the N-terminus to blue in the C-terminus. Green tubes show the motion associated with the principal components. The motion for PC2 does not involve significantly the interface residues, which is consistent with the lack of a stable interface for this dimer. (TIF) [file pone.0321064.s008.tiff]

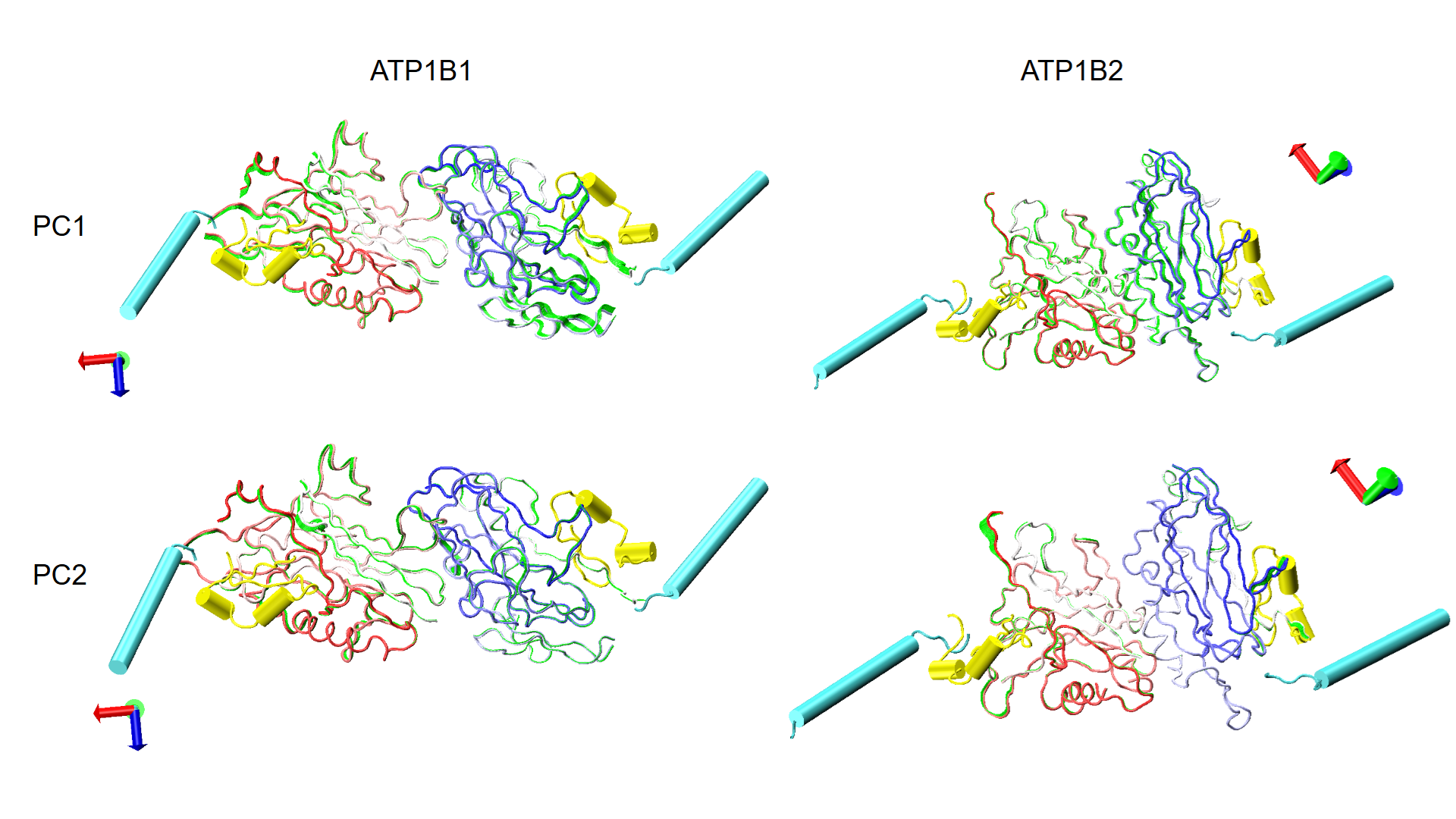

Supplement: S7 Fig — Coloring of the dimers and the associated motions as in Fig. 8. The position of alpha subunit L7-8 (shown in yellow) and the position of β subunit transmembrane span (shown in cyan) for β1 − β1, and the predicted positions of L7-8 (shown in yellow) and the transmembrane span for β2 − β2 (shown in cyan) were structurally aligned from the crystal structures used for the modeling (3WGU and 5YLU, respectively). The groove that would accommodate L7-8 is preserved in all cases. (TIF) [file pone.0321064.s009.tif]

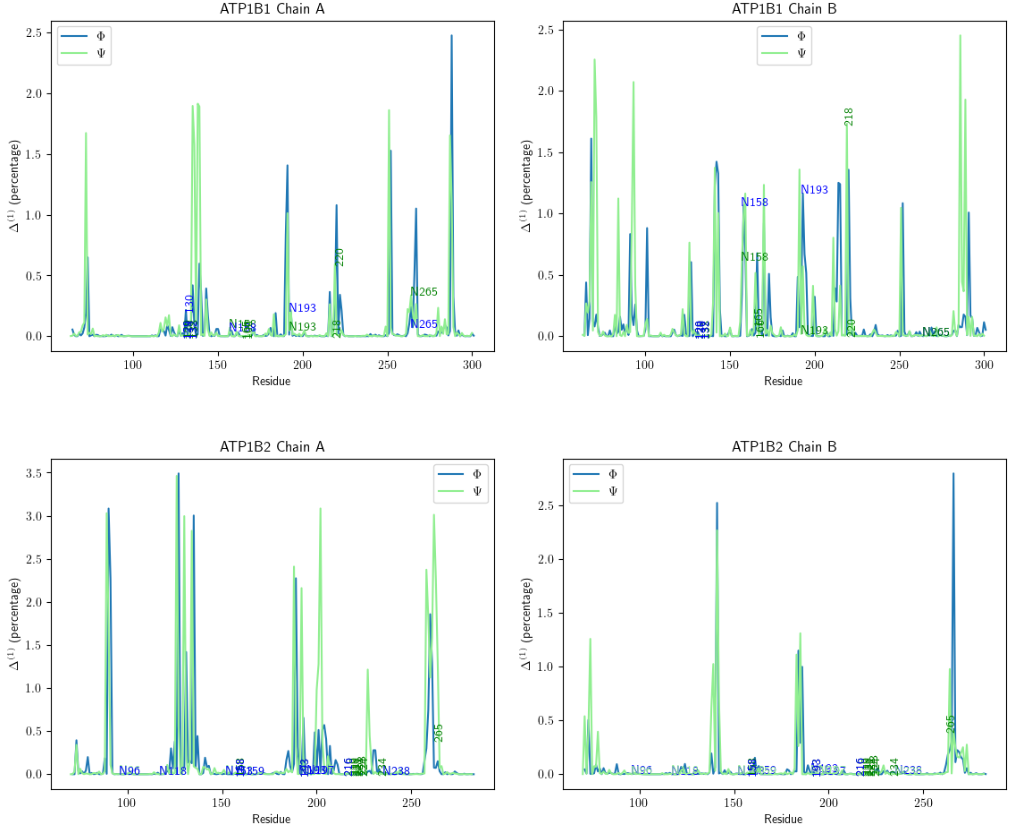

Supplement: S8 Fig — Vertical numbers indicate interface residues while horizontal numbers indicate glycosylated asparagines. For ATP1B1, Chain A, the highest peaks are in 5 Φ angles (Asn193, Asp222, Tyr254, Asp269, Arg290) and 9 Ψ angles (Val72, Glu135, Arg136, Asp138, Phe139, Asn193, Lys253, Asp289, Arg290), while for Chain B the highest values are found in 10 Φ angles (Tyr68, Arg143, Gly144, Glu145, Ser160, Ser195, Lys216, Arg217, Asp222, Tyr254) and 15 Ψ angles (Tyr68, Asp70, Arg71, Gln84, Asn93, Arg143, Glu145, Gly161, Gly172, Asn193, Lys221, Lys253, Lys288, Phe291, Gly293). The distribution of peaks for ATP1B2 is somewhat different, as for Chain A the highest peaks are in 8 Φ angles (Asn90, Leu91, Cys129, Arg133, Gln137, Asn193, Ala266, Asn267) and 17 Ψ angles (Glu89, Asn90, Val128, Gly132, Glu136, Ala192, Met196, Asp205, Glu206, Tyr231, Asn264-Thr270) while for Chain B the highest values are in 3 Φ angles (Leu143, Phe188,Asp272) and 5 Ψ angles (Gln74, Gly141, Leu143, Asn187, Tyr189). (TIF) [file pone.0321064.s010.tif]
