# Supplementary material for: In silico studies provide new structural insights into trans-dimerization of β1 and β2 subunits of the Na+, K+-ATPase
Source: PLoS One. 2025 Apr 29;20(4):e0321064. doi: 10.1371/journal.pone.0321064 (PMC12040271; doi:10.1371/journal.pone.0321064)
Supplement: Attachment — Submitted filename: Response to Reviewers R2.docx [file pone.0321064.s016.docx]

Manuscript number **PONE-D-24-40078R”**

**Response to Reviewers**

Estimated reviewers

We appreciate the time and efforts that you have dedicated to read and revise our manuscript again.

Here is our reply (in blue) to Reviewer #1.

Reviewer #1: Just a very minor request: for the stereo pictures in the supplement, they could be view at larger magnification if there was less white space between the images.

The images (Fig S3-S6) were corrected and they are now larger. Thanks.

Here is our reply (in blue) to Reviewer #2.

1. While the manuscript appears to be scientifically sound and likely acceptable for publication, the formatting issues in the submitted version are concerning. It reflects poorly on the submission process when such apparent errors are overlooked. Specifically, Greek letters are represented as empty boxes in the PDF, which makes it impossible to perform a thorough final review and provide a definitive recommendation in its current state.

We are very sorry for that. As you could notice by downloading the Word Document itself, not the PDF, the symbols and Greek letters are OK. This problem was generated by the system, when converting all uploaded files to one PDF document.

The corresponding author revised the document before submitting it, actually the system does not permit to continue if the author does not open the file and revise it. For a strange reason, we did not notice this error and we apologize for it.

2. Before resubmitting, I strongly urge the authors to double check that the manuscript is fully readable and that all formatting issues are resolved. See already on first page with several formatting errors.

It is double checked now and we hope you will get it as it is.

3. Also correct Line 285: use the Greek letter beta for the subunits as in other parts of the manuscript.

Thanks for your observation. It is now in Greek letter.
